# Moose Survival and Habitat‐Associated Risk of Endoparasites

**DOI:** 10.1002/ece3.72721

**Published:** 2025-12-29

**Authors:** Jennifer A. Grauer, Jacqueline L. Frair, Krysten L. Schuler, Manigandan Lejeune, David W. Kramer, Angela K. Fuller

**Affiliations:** ^1^ New York Cooperative Fish and Wildlife Research Unit, Department of Natural Resources and the Environment Cornell University Ithaca New York USA; ^2^ Department of Environmental Biology SUNY College of Environmental Science and Forestry Syracuse New York USA; ^3^ Wildlife Health Lab, Public and Ecosystem Health Department, College of Veterinary Medicine Cornell University Ithaca New York USA; ^4^ Department of Population Medicine and Diagnostic Sciences, College of Veterinary Medicine Cornell University Ithaca New York USA; ^5^ Roosevelt Wild Life Station SUNY College of Environmental Science and Forestry Syracuse New York USA; ^6^ U.S. Geological Survey, New York Cooperative Fish and Wildlife Research Unit, Department of Natural Resources and the Environment Cornell University Ithaca New York USA

**Keywords:** giant liver fluke, moose, parasite, survival

## Abstract

Parasite‐induced morbidity and mortality can alter the trajectories of incidental host populations. Yet, parasites rarely act in isolation and may be one of a multitude of biotic and abiotic stressors that collectively shape mortality risk in vertebrate populations. We quantified sources of mortality in a low‐density population of moose ( *Alces alces* ) in New York State and investigated factors including parasite infection, nutritional limitation, and thermal stress influencing mortality risk in calf moose. We observed high rates of annual survival (0.81–0.92) in adult (*n* = 25) and calf (*n* = 27) moose monitored 2015–2018 and 2022–2024, respectively. The majority of cause‐specific mortality was attributed to disease induced by giant liver fluke ( *Fascioloides magna* ; 75% in adults, 67% in calves). Calf mortality risk increased by 72% for every unit increase in giant liver fluke infection risk, measured as cumulative monthly proportion of wetlands used by moose, and decreased by 16% with each additional unit of nutritional energy available. The combination of flukes, coinfecting parasites, and available nutritional energy is important to calf survival in this population, highlighting the importance of managing multiple stressors for species conservation, although the effects are hard to disentangle given the high rates of survival observed. Identifying causes of mortality and mechanisms underlying increased mortality risk contributes to the continued conservation of moose in fluctuating populations and highlights the importance of managing parasite‐induced disease.

## Graphical abstract

We observed high rates of adult and calf moose survival in New York State, with mortality primarily due to disease associated with giant liver fluke infections. Calf mortality risk increased with increasing use of wetlands, where they can acquire giant liver fluke infections, and decreased with higher amounts of nutritional energy available.

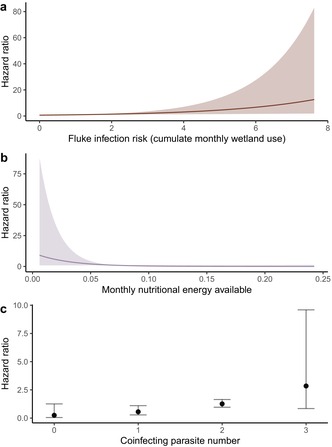

## 1 Introduction

Parasites and pathogens are capable of impacting host survival, frequently taking the place of predation as a mechanism of top‐down control (Raffel et al. 2008). Parasites can have direct consumptive effects on hosts as well as non‐consumptive effects on host body condition and mass, reproductive success, and behavior (Watson 2013). Hosts may suffer direct mortality, organ or tissue damage, and declines in function based on the severity and effects of parasite infections. Non‐consumptive effects include costs of avoiding parasites when cues of parasite presence are available (Doherty and Ruehle 2020) and chronic stress from maintaining parasite resistance or tolerance (Lafferty and Holt 2003). Understanding how mechanisms underlying these effects scale from the individual to the population level remains a critical challenge in wildlife health and conservation.

Parasites and their associated diseases impact the energy budgets of hosts through multiple pathways, affecting survival differently across age classes. For instance, males often invest less in immune defenses and exhibit lower parasite tolerance compared to females (Stephenson et al. 2016). Juveniles are particularly vulnerable as they develop their immune systems and are naïve to disease‐causing agents, as with the susceptibility of juvenile moose ( *Alces alces* ) to meningeal worm (*Parelaphostrongylus tenuis*) infections (Lankester 2018). Pathogens have similarly been a primary factor in bighorn sheep ( *Ovis canadensis* ) declines, where pneumonia impacts naïve members of the population and causes repeated years of low recruitment (Plowright et al. 2017). Conversely, adults can suffer cumulative damage from long‐term parasite exposure. Health impacts can scale to the population level by altering mortality rates, birth rates, abundance, and growth rates (Barber‐Meyer et al. 2008), particularly when impacts are unevenly distributed among different segments of the population (Hanley et al. 2022).

Parasites can directly cause host mortality, though the likelihood of mortality often depends on a combination of intrinsic and environmental factors that affect host immune function, stress levels, and body condition (Vollset 2019). Environmental conditions, such as extreme temperatures that shift body temperatures outside of optimal ranges, can induce physiological stress (Pörtner and Farrell 2008) and increase susceptibility to parasite‐induced mortality (Morley and Lewis 2014). Limited resources and poor body condition can also heighten the risk of mortality from parasitic infections (Washburn et al. 1991; Chin et al. 2017). Additionally, parasite coinfections can amplify stress; polyparasitism can result in synergistic effects that are more damaging than individual parasites alone (Thumbi et al. 2014). Specifically, the order and intensity of infections have been found to modulate host immune response, increase inflammatory responses, and exacerbate parasite pathologies (Supali et al. 2010). Furthermore, the interaction of environmental and intrinsic factors can magnify stress and alter host behaviors that increase risk of parasite infection or predation (Ruehle and Poulin 2020). These stressors are often prevalent at the edges of a species' range, where populations frequently face harsher thermal and nutritional conditions compared to those in the core of their range (Rehm et al. 2015).

The estimation of demographic vital rates, and disentangling of mechanisms limiting them, is a critical component of successful wildlife management and conservation. While adult survival is often the most elastic and sensitive vital rate for long‐lived mammals (Heppell et al. 2000), contributing the most to population growth, managers often have limited ability to increase this rate when survival is already high. Other rates, such as survival of younger age classes or fecundity, may be important limiting factors for population growth and serve as more tractable targets for management (Johnson et al. 2010). For long‐lived species, juveniles are often the age class most susceptible to mortality; their survival is typically the most variable and can have disproportionate effects on population dynamics (Coulson et al. 2005). Subsequently, the quantification of juvenile survival and mechanisms negatively impacting survival can be critical to conserving local populations when other demographic rates are high.

Moose populations along the southern limits of their North American range have experienced fluctuations and repeated declines (Wattles and DeStefano 2011). While some populations persist despite high temperatures (Wattles et al. 2018) and have expanded into areas of suboptimal forage (Maskey Jr and Sweitzer 2020), many contend with parasites and pathogens that impact survival. Moose are particularly vulnerable to infections such as meningeal worm and giant liver fluke ( *Fascioloides magna* ) due to spatial overlap with white‐tailed deer ( *Odocoileus virginianus* ), which can lead to lethal outcomes and regional population declines (Lankester et al. 2007). Moose in Minnesota have experienced high juvenile mortality due to the concomitant stressors of predators, parasites, malnutrition, and climate (Murray et al. 2006), while populations in New England, which are largely predator‐free, suffered high mortality primarily from winter tick ( *Dermacentor albipictus* ; Jones et al. 2019; Ellingwood et al. 2020). Coinfections can heighten vulnerability to additional parasites and pathogens (Murray et al. 2006; Pekins 2020) and have contributed to low calf survival rates in Vermont (DeBow et al. 2021). In the same region, the combination of nutritional status and physiological impacts of parasite infection in adult moose has led to decreased reproduction and calf recruitment (DeBow et al. 2022). Moose additionally experience metabolic stress from ambient temperatures exceeding upper critical thresholds of −5°C in winter and 14°C in summer (Lenarz et al. 2009). Moose adjust behaviorally by increasing use of thermal refuges (e.g., mature conifer forests and wetlands; Alston et al. 2020), though space use could potentially alter moose risk of acquiring certain parasite infections if it increases overlap with deer and use of infected habitats.

Giant liver flukes rely on white‐tailed deer as primary hosts and aquatic snails as intermediate hosts to complete their life cycles. Moose are dead‐end hosts to giant liver fluke and become infected when they ingest fluke metacercariae that have encysted on aquatic vegetation (Králová‐Hromadová et al. 2016). These infective metacercariae develop from free‐swimming cercariae released from infected aquatic snails, posing a risk to alternative hosts typically from May through October (Csivincsik et al. 2023), with prevalence rising as hosts spend more time in wetlands (Normandeau et al. 2020). While white‐tailed deer experience minimal pathology from liver fluke infections, moose can suffer severe damage, including liver and lung necrosis as flukes migrate and pseudocysts are created through host immune response (Pybus 2001). Evidence has indicated a range of pathology in moose, spurring debate on fluke infection as a direct cause of mortality (Lankester and Foreyt 2011; Wünschmann et al. 2015). Yet, flukes have repeatedly been identified as the primary cause of mortality in moose and other incidental hosts, such as domestic ruminants, that contend with heavy infections and additional stressors (Pybus et al. 2015). The deleterious effects of infection with liver flukes, among other parasites, could constitute one of several interacting factors that limit moose survival along their range edges (Escobar et al. 2019), although the extent of this in the northeast United States is unknown.

Moose recolonized New York State in the 1980's where, despite apparently high habitat availability within the Adirondack Park, the population has persisted at lower densities compared to other northeastern states (Hinton et al. 2022). Black bears ( *Ursus americanus* ) and coyotes ( *Canis latrans* ) may consume young moose calves, though predation in the region is rare (Benson et al. 2017; Wattles and DeStefano 2011). In contrast, considerable overlap with white‐tailed deer has resulted in moose infection with meningeal worm and giant liver fluke. Opportunistic sampling has indicated that the primary mortality threats to New York moose include vehicle collisions and death from meningeal worm and giant liver fluke infection (Grauer 2024). Environmental factors such as limited nutritional energy content (Peterson et al. 2020), other parasitic infections (Grauer 2024), and warming temperatures (Teitelbaum et al. 2021) potentially place additional physiological stress on moose. Understanding the cumulative and interactive effects of these stressors on moose survival is needed to help ensure persistence of this marginal population into the future.

Our objectives were to quantify adult and calf survival rates of moose in the Adirondack Park, New York, and understand factors influencing mortality risk of moose calves. We aimed to identify cause‐specific sources of mortality and determine the degree to which parasites or interacting stressors are influencing mortality rates in this population. Given the low density of moose in New York (Hinton et al. 2022) and the low prevalence of winter tick observed (Grauer 2024), we expected New York moose to exhibit higher calf survival rates relative to neighboring populations that have experienced winter tick epizootics. We further hypothesized that calf mortality risk would increase with: habitat use that exposes moose to giant liver fluke infection; high temperatures that induce metabolic stress; coinfection with additional endoparasites; nutritional energy limitations; and interactions among these factors.

## 2 Materials and Methods

### 2.1 Study Area

We monitored moose survival in the Adirondack Park in northern New York, USA from January 2015 to May 2018 (adults) and January 2022 to August 2024 (juveniles). The Adirondack Park was established in 1885 and consists of approximately 23,470 km^2^ of publicly and privately owned land in northern New York State. Over 61% of the Park consists of state‐owned public land managed as “forever wild” forest preserve, which precludes development and resource extraction (New York State Constitution, Article XIV). Cover types consist of temperate deciduous forests, northern boreal forests, lakes, and wetlands, and vary across elevations ranging from 30 to 1600 m above sea level. Primary tree species in the area include red and sugar maple ( *Acer rubrum* , *A. saccharum* ), yellow and paper birch ( *Betula alleghaniensis* , *B. papyrifera* ), American beech ( *Fagus grandifolia* ), white pine ( *Pinus strobus* ), and balsam fir ( *Abies balsamea* ; Peterson et al. 2022). Habitat within the Park has been estimated to support a population of approximately 760 moose (SD = 428; Kramer et al. 2022), and empirical estimates indicate the population is approaching that level (656 moose, 95% CI = 501–859; Hinton et al. 2022). Currently, no hunting of moose is allowed in the state.

### 2.2 Live Capture and Parasite Assessment

We captured moose during January each year in 2015–2017 and 2022–2023. Capture was conducted by a contracted company (Native Range Capture Services, Elko, Nevada, USA) consisting of helicopter pilots, handlers, and a veterinarian. Capture in 2015–2017 targeted adult female moose (≥ 2 years old) whereas capture in 2022–2023 targeted juveniles (yearlings ~1 year 8 months old, and calves ~8 months old). Given sufficient snow, net‐gunning was used to restrain moose, whereas under low snow conditions moose were darted and immobilized using 0.03 mg/kg of thiafentanil. All moose were restrained and blindfolded. Chemically immobilized moose received reversal injections of 0.6 mg/kg of naltrexone (Arnemo and Kreeger 2018). Capture in 2015–2017 followed the requirements of the State University of New York, College of Environmental Science and Forestry Institutional Animal Care and Use Committee (IACUC; protocol 140901), and capture in 2022–2023 followed requirements of the Cornell University IACUC (protocol 2021‐0006). Moose were fitted with either BASIC Iridium Track M 3D collars (Lotek Inc. Newmarket, Ontario, Canada), TGW‐4670‐3 collars (Telonics, Mesa, Arizona, USA), or VERTEX Lite‐3D radio collars (VECTRONIC Aerospace GmbH, Berlin, Germany). Collars were programmed to collect global positioning system (GPS) location data every 2 h and emit a very high frequency (VHF) beacon for 12 h a day between 0600 h and 1800 h (EST). Juvenile collars (VERTEX Lite‐3D) were retrofitted to expand with growing neck circumference according to methods used by Musante et al. (2010) and Jones et al. (2017). Each collar was programmed with an internal drop‐off mechanism timed to release 130 weeks after deployment.

During capture, we collected blood and fecal samples to assess parasite exposure and infection. Blood was drawn from the jugular vein using an 18‐gauge needle and 30 mL syringe and stored in clot activator vacutainer tubes. We centrifuged blood samples at 1100 *g* for 15 min within 1–6 h of collection to separate serum, which was transferred to cryovials and stored frozen until analysis. We tested serum for *P. tenuis* antibodies using an enzyme‐linked immunosorbent assay (ELISA) diagnostic test at the University of Tennessee College of Veterinary Medicine (Richards et al. 2023). We also tested for *Neospora caninum* infection using an indirect fluorescent antibody titer at the Animal Health Diagnostic Center (AHDC) at Cornell University (Ithaca, NY). Fecal samples were collected, stored frozen at −18°C, and analyzed for parasite eggs using fecal flotation (Flukefinder Visual Difference, Moscow, Idaho, USA) and larvae using a modified Baermann technique (Forrester and Lankester 1997) at the AHDC. We identified nematode eggs from the genus *Nematodirus* and tapeworm eggs from the genus *Moniezia* during fecal flotation and categorized the presence of each at the genus level. Giant liver flukes fail to complete their life cycles and do not shed eggs in moose feces (Murray et al. 2006) and there is no currently available diagnostic assay to detect giant liver flukes in serum; therefore, we were unable to quantify giant liver fluke prevalence at capture.

### 2.3 Survival and Cause‐Specific Mortality

Collars were programmed to emit a mortality signal when moose were stationary for at least 6 h. Once a mortality signal was received, New York State Department of Environmental Conservation (NYSDEC) staff located the animal within 72 h to determine if the animal was dead and collect the carcass for examination. Mortalities were necropsied by staff pathologists and veterinarians at the NYSDEC Wildlife Health Unit (WHU; Delmar, New York) to assess cause of death and parasite infection status. If the entire moose carcass could not be collected, biologists conducted a field necropsy to assess cause of death and collected the head, lungs, liver, and heart for inspection at the WHU. Mortalities were classified as caused by giant liver fluke if flukes, cysts, or lesions causing extensive damage (≥ 60% on average) to the liver or lungs were identified and other discernable sources of mortality were lacking. Mortalities were categorized as trauma if moose experienced wounds that led to death or euthanasia by staff biologists. When no clear causes of death were identified or the decomposition status of moose precluded examination, the cause of death was unknown.

We estimated survival probabilities using a modified Kaplan–Meier estimator (Pollock et al. 1989) to allow for staggered entry across multiple years of capture. Animals were included in the analysis from their date of capture. We excluded animals that died within 1 month of capture from all statistical analyses to remove individuals that may have died from capture‐related myopathy (Jones et al. 2019). Animals were tracked given a status of “1” for death upon the date of mortality alert, or censored (“0”) on dates when collars failed or successfully dropped off the animal.

We summarized cause‐specific mortality and quantified proportional contribution of different causes of mortality using nonparametric cumulative incidence functions (Satagopan et al. 2004). Survival and cumulative incidence analyses were conducted separately for adults and calves and included no additional covariates considering limited sample sizes. All analyses (Grauer and Fuller 2025) were conducted in program R (R Core Team 2021) using packages survival (Therneau 2024), tidycmprsk (Sjoberg and Fei 2024), and ggsurvfit (Sjoberg et al. 2024).

### 2.4 Modeling Monthly Calf Mortality

We investigated the drivers of monthly calf mortality using Cox proportional hazards analysis with time‐varying covariates (Bellera et al. 2010). We selected the Cox proportional hazards method to measure the instantaneous rate of mortality events with the flexible inclusion of a nonparametric distribution for the hazard function (Fox and Weisberg 2002). Due to the small number of adult mortalities and conflation of age with sampling period, we only included calves in this analysis and did not investigate factors important for adult mortality risk.

We included biological covariates hypothesized to influence the probability of moose being infected with or succumbing to infections from giant liver fluke. We characterized fluke infection risk by quantifying the proportion of areas around moose relocations where moose could be exposed to infective metacercariae of giant liver fluke. Specifically, we quantified land cover within a circular buffer (115 m radius) around each moose relocation based on mean step length ($\bar{x}$ = 112.68, SD = 43.08) between all calf 2‐h relocations. We calculated the proportion of each buffer consisting of wetland land cover types (Ferree and Anderson 2013) and then averaged the mean proportion of wetland by month. The mean proportion of wetland characterized infection risk May through October, when fluke metacercariae were available on the landscape to infect moose (Csivincsik et al. 2023); risk was set to zero November through April assuming metacercariae were not available in wetlands during these months. Overlap of buffers allowed for added weight to longer periods of time spent (multiple relocations) in wetlands, which we expected to increase mortality risk. We expected risk to increase over time, where cumulative exposure could increase the number of flukes accrued, intensities of infection, and ultimate mortality from infection. Thus, we summed the mean proportion of wetlands used in consecutive months to characterize cumulative risk of acquiring giant liver fluke infections.

To test whether nutritional limitations were contributing to moose mortality, we quantified the amounts of energy available to moose within the 115‐m radius buffers around moose relocations. We calculated this as the energy animal use days per km^2^ estimated by Peterson et al. (2022) for conifer forest, upland and lowland deciduous and mixed forest, wooded wetland, open wetland, and regenerating forest (Table S1). Animal use days varied by season (summer = May–October, winter = November–April) to account for variable energetic needs of adult female moose and the digestible energy and crude protein seasonally available in vegetation (Peterson et al. 2022). We used the predicted regenerating forest cover map developed by Kramer et al. (2022), reflecting intermediate and overstory timber removal occurring from 2003 to 2018, to characterize regenerating forest and layered this on top of The Nature Conservancy Terrestrial Habitat Map (Ferree and Anderson 2013) to characterize remaining habitat cover types. We then calculated mean proportions of cover types used in each month. We multiplied these mean monthly proportions by the energy available in each cover type in each season, then summed values to characterize the mean energy available to each moose in a given month. We included a second covariate of cumulative nutritional availability over time by adding consecutive monthly values to characterize nutritional condition over the course of the study.

To investigate whether thermal stress contributed to calf mortality, we first calculated mean daily maximum temperatures experienced by each moose by overlaying ambient temperature data (PRISM Climate Group, n.d.) with moose relocations. We then averaged values over each month and calculated the difference between monthly mean maximum temperatures and season‐specific thresholds known to cause metabolic stress to moose (McCann et al. 2013). Specifically, we used the threshold of −5°C in winter months (November–March) and 14°C for other seasons (April–October; Wattles et al. 2018). We additionally explored the higher temperature thresholds of 0°C in winter months and 20°C in other seasons (Wattles et al. 2018), though this did not alter our subsequent modeling results.

Parasite coinfection was included as the number of coinfecting parasite species detected using the *P. tenuis* ELISA, *N. caninum* antibody test, and fecal flotation tests. Each moose had a single value for coinfecting parasites held constant across each month of the study; coinfection was recorded only at the time of capture and not updated over time.

All covariates were centered and scaled. We checked for correlation between covariates and included only one covariate from pairs where Pearson's correlation |*r*| > 0.6 (Dormann et al. 2013). We included uncorrelated covariates in a full model and tested for the assumption that baseline hazard for each covariate was constant over time. This assumption was violated if the test for the Cox regression model fit for any covariate or the global model was significant (*p* < 0.05) and covariates were not independent of time (Harrell Jr. and Harrell 2015). We constructed a set of 16 candidate models using the covariates: fluke infection risk, temperature, nutritional energy, and coinfecting parasites. Models included univariate models with each covariate alone, two‐way and three‐way additive combinations and two‐way interactions between each covariate and fluke risk, a null model, and a full model including each covariate additively and the aforementioned interactions. We used Akaike's Information Criterion adjusted for small sample sizes (AIC_c_) to evaluate candidate models, determined competing models as those with ΔAIC_c_ < 2, and selected the top‐ranked model (lowest AIC_c_) for inference (Anderson and Burnham 2002). We predicted hazard ratios for statistically significant covariates included in the top model, varying the covariate of interest while holding other included covariates at their mean values. Similarly, we used Aalen's additive regression model (Aalen and Scheike 2014; survival package aareg function) with covariates of the top‐ranked Cox model to examine the cumulative hazard of each covariate over time.

## 3 Results

### 3.1 Moose Capture and Survival

We captured 25 adult moose between 2015 and 2017 (Table 1) including two males and 23 females. We captured 34 juvenile moose between 2022 and 2023 including two yearling females and 32 calves (18 male, 14 female). Moose from 2015 to 2017 were monitored until May 2018 and moose from 2022 to 2023 until August 2024. All moose in 2015–2017 and 2022 were captured by netting while moose in 2023 were captured by darting. The two yearlings were censored from all subsequent analyses due to small sample size for this stage. Five calves died within 1 month of capture and were censored from subsequent statistical analyses (Jones et al. 2019). These included three calves that died in 2022 with cervical vertebrae fractures (potentially caused by netting in low, hard‐pack snow conditions) and two moose in 2023 that died from septicemia. Cases with septicemia could not conclusively be attributed to either giant liver fluke infestations or capture‐related causes. No other signs of capture‐related myopathy were observed.

**TABLE 1:** Summary of captures and causes of mortality for radio‐collared moose ( *Alces alces* ) in the Adirondack Park, New York, USA, between 2015–2017 and 2022–2024.

Year	Age at capture	Sex	Captured	Deaths	Censored	Cause of death		
						Liver fluke	Trauma	Unknown
2015	Adult	F	9	1			1	
		M	2					
2016	Adult	F	9	3		3		
		M						
2017	Adult	F	5					
		M						
2022	Calf	F	7	1	1	1		
		M	6		2			
2023	Calf	F	7	3		3		
		M	12	3	2	2		1
2024	Calf	F		3			1	2
		M		2		2		
Total			59	16	5	11	2	3

*Note:* Included are the number of females (F) and males (M) captured, the number of collared moose deaths, individuals censored from subsequent analyses, and causes of death including giant liver fluke ( *Fascioloides magna* ), trauma, and unknown sources. Adults were those animals > 2 years old at capture and calves were approximately 8 months old at capture and transitioned to yearlings after 4 months.

We observed four mortalities of collared adults and 12 mortalities of collared calves. Adult mortalities were attributed to trauma (*n* = 1; January) and giant liver fluke infections (*n* = 3; January, June, August). Calf mortalities included trauma (*n* = 1; June), giant liver fluke (*n* = 8; March–June, August, October), and unknown causes of death (*n* = 3; June, August, September). In two cases where the cause of death could not be determined, carcasses could not be recovered before decomposition precluded assessment. In the third case, no discernible causes of death were determined from field or laboratory necropsy.

**FIGURE 1:**
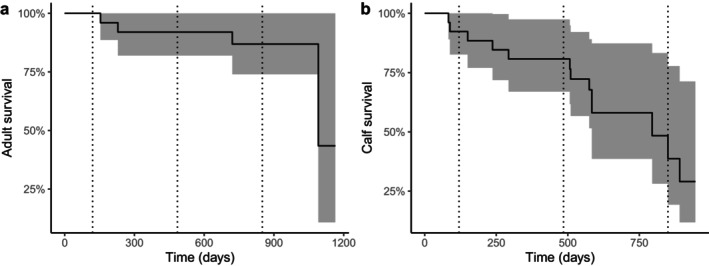
Kaplan–Meier survival estimates and 95% confidence intervals (shaded) for adult (a; *n* = 25) and calf (b; *n* = 27) moose ( *Alces alces* ) in the Adirondack Park, New York, USA between 2015–2018 and 2022–2024, respectively. Dotted vertical lines indicate 120, 485, and 850 days following capture, representing the approximate birthdates of moose in early May of each year.

**FIGURE 2:**
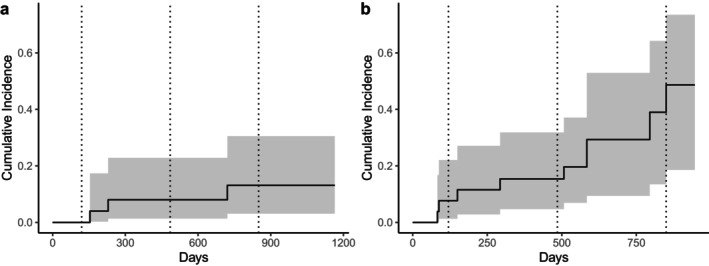
Cumulative incidence of adult (a; *n* = 25) and calf (b; *n* = 27) moose ( *Alces alces* ) succumbing to giant liver fluke ( *Fascioloides magna* ) infections in the Adirondack Park, New York, USA. Shaded regions represent 95% confidence intervals. Dotted vertical lines indicate 120, 485, and 850 days following capture, representing the approximate birthdates of moose in early May of each year.

Estimated annual adult survival (Figure 1a; *n* = 25) was 0.92 (95% CI [0.82, 1.00]). Calf winter survival (Figure 1b; *n* = 27) for the first 4 months following capture until individuals reached approximately 1 year of age was also 0.92 (95% CI [0.83, 1.00]). For those calves that survived to the yearling stage (*n* = 25), annual survival dropped to 0.81 (95% CI [0.67, 0.97]). The cumulative incidence for adult moose succumbing to giant liver fluke (Figure 2a) was 0 at 120 days, 0.08 at 485 days, and 0.13 at 850 days in the study, approximating incidence at annual birthdates in early May of each year. For calves, the risk of succumbing to fluke infection (Figure 2b) was 0.08 at 120 days, 0.15 at 485 days, and peaked at 0.49 at 850 days.

### 3.2 Modeling Monthly Calf Mortality

The monthly mean proportion of wetlands used by calf moose May–October ranged from 0 to 0.96 ($\bar{x}$ = 0.18, SD = 0.20). Resulting fluke risk, measured as cumulative monthly mean proportion of wetlands used, varied from 0 to 7.62 ($\bar{x}$ = 1.2, SD = 1.45). Mean monthly nutritional energy available ranged from 0.01 to 0.24 ($\bar{x}$ = 0.07, SD = 0.05). Maximum ambient temperatures during the study ranged from −6.3°C to 26.9°C, resulting in temperature differences from thresholds of −5°C and 14°C ranging from −4.6 to 12.97 ($\bar{x}$ = 5.92, SD = 4.15).

Numbers of *Nematodirus* varied from 0 to 13 eggs per gram of feces ($\bar{x}$ = 3.45, SD = 3.42), and numbers of *Moniezia* ranged from 0 to 76 eggs per gram of feces ($\bar{x}$ = 5.28, SD = 15.43). No lungworm larvae were detected in fecal samples. Twenty‐two moose were infected with *Nematodirus* and seven moose were infected with *Moniezia*. *Parelaphostrongylus tenuis* infection was detected in one calf, and *N. caninum* was detected in 18 calves. Consequently, the number of infecting parasite species ranged from zero to four, including *P. tenuis* , *N. caninum*, and the two parasite genera identified from fecal flotation. No calves were infected with four parasite species; four calves were infected with three species, 13 with two species, 10 with one species, and two with zero species.

**FIGURE 3:**
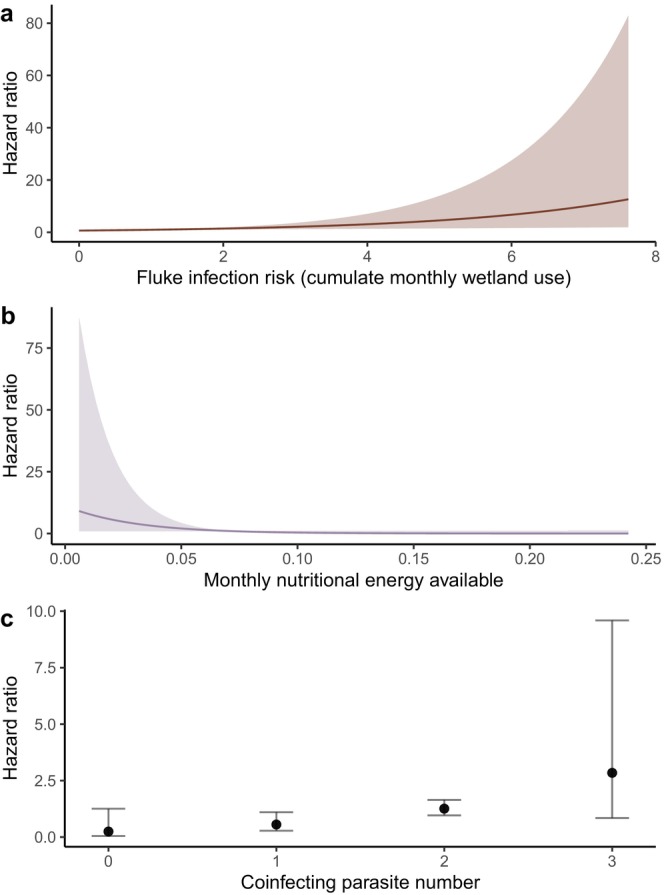
Predicted hazard ratios for fluke infection risk (a), nutritional energy available (b), and number of coinfecting parasites (c) for calf moose ( *Alces alces* ; *n* = 27) in the Adirondack Park, New York, USA from 2022 to 2024. Fluke risk was quantified as the cumulative proportion of wetlands used per month where moose can acquire giant liver fluke ( *Fascioloides magna* ) infections, nutritional energy as the energy content of mean monthly habitat proportions available to moose, and coinfecting parasites including *Parelaphostrongylus tenuis*, *Neospora caninum*, *Moniezia* spp., and *Nematodirus* spp. Shaded region and error bars represent 95% confidence intervals from the top Cox proportional hazards model that included the three covariates additively.

**FIGURE 4:**
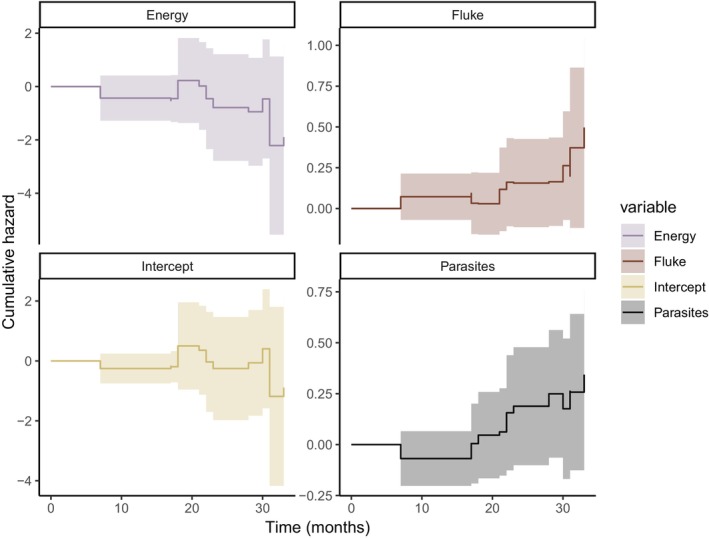
Cumulative hazard over time in months for calf moose ( *Alces alces* ; *n* = 27) in the Adirondack Park, New York, USA from 2022 to 2024. Lines are estimates and shaded regions 95% confidence intervals from the Aalen's additive regression model that included fluke risk (fluke), coinfecting parasites (parasites), and nutritional energy (energy) available. Fluke risk was quantified as the cumulative proportion of wetlands used per month where moose can acquire giant liver fluke ( *Fascioloides magna* ) infections, coinfecting parasites including *Parelaphostrongylus tenuis*, *Neospora caninum*, *Moniezia* spp., and *Nematodirus* spp., and nutritional energy as the energy content of mean monthly habitat proportions available to moose.

Fluke risk and cumulative nutrition were collinear (*r* = 0.79) because of the cumulative structure imposed; we retained cumulative fluke risk and monthly nutrition to avoid collinearity in subsequent modeling. To determine whether our choice of including cumulative fluke risk rather than cumulative nutrition impacted model inference, we explored an alternative model set with the effects of cumulative nutrition and monthly fluke risk in addition to remaining covariates (Appendix S1). This model set followed the identical structure of 16 candidate models and was ultimately less informative (lower overall AIC_c_ values; Tables S2 and S3) than the model set including cumulative fluke and monthly nutrition. All remaining covariates had a correlation of |*r*| < 0.25. The global additive model including fluke risk, coinfecting parasites, temperature, and nutritional energy met assumptions for independence of the overall model and each covariate with time (global *Χ* ^2^ = 1.18, *p* = 0.88). Four of the 16 models had ΔAIC_c_ < 2 (Table 2), and the top‐ranked model included the additive effects of fluke risk, coinfecting parasites, and nutritional energy; however, only the effect of fluke risk was significant at an alpha level of 0.05 (Table 3). The null model had the lowest support (ΔAIC_c_ = 5.61), with univariate models having slightly more support but with ΔAIC_c_ > 3. The three competing models all included fluke risk, with differing additive combinations of nutrition, coinfecting parasites, and temperature. Fluke risk was statistically significant in all of these models, and nutritional energy was significant at the alpha level of 0.10 in two models. The global model included no significant covariate effects. Fluke risk (*β* = 0.54, *p* = 0.008) and coinfecting parasites (*β* = 0.56, *p* = 0.092) were positively associated, and available nutritional energy (*β* = −1.84, *p* = 0.056) negatively associated with calf mortality. Specifically, moose experienced a 72% increase in mortality risk for each unit increase in fluke risk (hazard ratio 95% CI [1.15, 2.58]), a 75% increase in mortality risk for each additional coinfecting parasite species (hazard ratio 95% CI [0.91, 3.35]), and a 16% decrease in mortality risk for every increase in nutritional energy available (hazard ratio 95% CI [0.02, 1.05]). For predicted hazard ratios (Figure 3), this translated to hazard ratios over 1.0 for moose given fluke risk above 1.2 and at least two coinfecting parasites. Hazard ratios were approximately 2.3 times higher for moose infected with three coinfecting species compared to those with two coinfecting species. Hazard ratios exceeded 8.18 for moose with the top 10% highest cumulative proportions of wetlands available. Hazard dropped below one for moose when monthly nutritional energy available exceeded 0.075. In the test of cumulative hazard over time using additive combinations of fluke risk, parasite number, and nutritional energy (Figure 4), no hazards varied significantly over time (*Χ* ^2^ = 6.01, *p* = 0.11).

**TABLE 2:** Model selection of Cox proportional hazards for monthly survival of calf moose ( *Alces alces* ) in the Adirondack Park, New York, USA.

Model	*k*	AIC_c_	ΔAIC_c_
fluke + parasite + nutrition	4	61.229	0
fluke + nutrition	3	62.170	0.941
fluke + parasite + nutrition + temp	5	62.571	1.342
fluke + temp + nutrition	4	62.701	1.472
fluke*nutrition	4	63.531	2.302
fluke + parasite	3	63.782	2.553
fluke + temp	3	63.839	2.610
fluke + parasite + temp	4	63.997	2.768
fluke	2	64.547	3.318
fluke*temp	4	65.048	3.819
fluke*parasite	4	65.235	4.005
nutrition	2	66.082	4.853
parasite	2	66.280	5.051
full	8	66.554	5.324
temp	2	66.673	5.443
null	1	66.839	5.610

*Note:* We explored the cumulative proportion of wetlands used per month, approximating risk of moose acquiring giant liver fluke (fluke; *Fascioloides magna* ) infections, the monthly nutritional energy (nutrition) available to moose, number of coinfecting parasites (parasite), and differences between mean ambient temperature maximums per month and stress‐inducing thresholds (temp). Included are the additive and interactive models tested with their number of parameters (*k*), Akaike's information criterion corrected for small sample size (AIC_c_), and difference in AIC_c_ (ΔAIC_c_).

**TABLE 3:** Parameter values for top Cox proportional hazards model of calf moose ( *Alces alces* ) mortality in New York, USA from 2022 to 2024 that included fluke risk, parasite number, and nutritional energy available.

Covariate	*β*	Exp(*β*)	95% CI	*p*
Fluke risk	0.54	1.72	1.15–2.58	0.008
Parasite number	0.56	1.75	0.91–3.35	0.092
Nutritional energy	−1.84	0.16	0.02–1.05	0.056

*Note:* Fluke risk was quantified as the cumulative proportion of wetlands used per month where moose can acquire giant liver fluke ( *Fascioloides magna* ) infections, parasite number as the number of detected coinfecting parasite species, and nutritional energy as the energy content of mean monthly habitat proportions available to moose. Included are the coefficient (*β*) values, hazard ratio (Exp(*β*)), 95% confidence intervals (CI), and statistical significance (*p*) for each included covariate.

## 4 Discussion

Giant liver flukes pose the greatest parasitic threat to moose in New York, with additional coinfecting parasites potentially contributing to and nutritional condition buffering against calf moose mortality. Our findings are consistent with parasite coinfections being the norm rather than the exception in wild populations (Lello et al. 2004), and within‐host interactions being a potentially significant component of host fitness (Jolles et al. 2008). We observed that mortality risk increased with greater cumulative wetland use, which assumedly increased potential fluke infection risk, and was possibly exacerbated when moose were simultaneously infected with two or three additional parasites and had low levels of nutritional energy available. Our modeling provides strong evidence that giant liver fluke infections can be deadly for moose and raises important questions for further research on the additional stress from multiple parasite infections and buffering effect of adequate nutritional energy.

Although winter ticks have impacted body condition and led to high mortality of moose in nearby New England states (Jones et al. 2019; Ellingwood et al. 2020), internal parasites appear to fill a similar, albeit diminished, role in New York. Adult annual survival in our region (92%) was comparable to populations in Ontario, Canada (90%; Murray et al. 2012), Vermont (88%; DeBow et al. 2021), and New Hampshire (87%; Ellingwood et al. 2020) in years without winter tick epizootics. Calf winter survival in New York (92%) was higher than comparable regional estimates in years with high winter tick mortality (49% in Vermont; DeBow et al. 2021; < 40% in New Hampshire and Maine; Jones et al. 2019). Estimates in non‐epizootic years of 69% calf survival in New Hampshire (Ellingwood et al. 2020) and 60% in Vermont (DeBow et al. 2021) were still significantly lower than New York estimates, though these estimates reflect the full annual period of calf survival, whereas our calves were only monitored in the 4 months prior to becoming yearlings. Yet, Adirondack calves that advanced to yearlings had low survival (81%) relative to neighboring states, where yearling survival was comparable to that of adults (Ellingwood et al. 2020). Interestingly, calves in New England experience high rates of lungworm infection (70% in Vermont; DeBow et al. 2021; 87% in New Hampshire and Maine; Jones et al. 2019), but we found no lungworm infections in our captured moose of the same age and comparatively low numbers in necropsied moose (5%; Grauer 2024). Rather, prevalence of giant liver fluke was high (94%) in collared calf mortalities and > 50% in opportunistically necropsied juvenile moose in New York (Grauer 2024).

Giant liver fluke infections are common in some moose populations and, despite controversy over their impact, have increasingly been recognized as direct sources of mortality (Carstensen et al. 2018; Shury et al. 2019). Quantifying the cumulative proportion of wetlands used during periods of fluke infection risk allowed us to explore both exposure potential for moose and the risk of accumulating fluke infections over time. Although the risk of infection is likely dependent on a number of factors, such as biotic conditions important for aquatic gastropod population dynamics and survival of free‐swimming cercariae, here cumulative wetland use during infective periods functioned as a proxy for moose risk of infection with giant liver fluke. Similar research found intensities of giant liver fluke infections in elk ( *Cervus elaphus canadensis* ) were correlated with wetland use (Normandeau et al. 2020), and fine‐scale wetland imagery has been proposed for spatial modeling of similar fluke species (De Roeck et al. 2014). Still, land cover types have translated poorly to predicted areas of gastropod host presence (Vannatta and Moen 2016), and remotely sensed habitat layers often lack information on ephemeral wetlands or small areas of standing water where infected gastropods could occur and moose acquire giant liver fluke infections. Thus, by only including satellite‐based assessments of wetland cover, our estimate of fluke infection risk may underestimate total risk to moose. Additionally, infection risk may increase with climate change as moose increase their use of wetlands to cope with higher temperatures, and wetter conditions and greater spatial overlap with white‐tailed deer could lead to higher parasite abundance on the landscape (Maskey Jr 2008). Other factors such as white‐tailed deer density may be more informative than wetland availability (Peterson et al. 2013), though fine‐scale deer density is lacking for this region and coarse indices were previously negatively associated with fluke infection in moose (Grauer 2024). Our inclusion of wetland use could overlap with other factors important for moose survival, including the provision of aquatic vegetation during the growing season (Tischler et al. 2019) and wetland use for thermoregulation (Thompson et al. 2021). Nevertheless, our findings contribute to the mounting evidence that giant liver flukes tangibly impact moose mortality, and that wetland use is an important consideration for understanding infection dynamics of incidentally infected cervid hosts (Kasny et al. 2012; Pybus et al. 2015).

Given the preponderance of giant liver fluke infections, the survival differences we observed between calves and subsequent yearlings may reflect infection dynamics, namely, the time needed to accumulate heavy infestations and experience the damaging effects of infection (Pybus 2001). Because our sampling confounded moose age with capture year, we could not discern whether calves that survived to become yearlings were indeed the most greatly affected, nor whether adults possessed greater tolerance or an enhanced ability to mitigate the deleterious effects of infection (Råberg 2014). Nevertheless, infection rates remain high in adulthood, with adults succumbing to heavy infestations that damage > 60% of the host liver (Grauer 2024; Escobar et al. 2019). Hence, relatively common fluke infections, in combination with years of poorer nutrition or additional coinfections, may contribute jointly to adult mortality. The limited number of adult mortalities in this study prevented deeper investigation of mechanisms driving adult mortality, and conflation of ages with capture seasons prevented our comparison of parasite infections with moose age class. Additionally, the small sample size of captured calves and number of mortalities limited our exploration of more complex survival models. Despite our striving to explore the additive and interactive effects of included factors, we could only reliably make inference from univariate models. Specifically, while the impact of giant liver flukes is supported, the additive effects of nutrition and coinfecting parasites are weaker and reflect trends that were not replicated in models with those factors alone. Thus, we cannot rule out the effects of other factors, including temperature, or specify the direction or magnitude of any interactions between these factors. While sampling of low‐density populations is financially and logistically challenging, larger sample sizes for both calves and adults over additional years of capture would provide increased statistical power and allow for further exploration of factors important for fluke transmission to moose, parasite coinfection, and nutritional stress over time.

The presence of coinfecting parasite species featured repeatedly in competitive models and was marginally significant to calf mortality risk. Parasite richness can impose cumulative and synergistic health effects on hosts, impacting wildlife populations and the evolution of parasite defenses (Bordes and Morand 2011). Although typically not considered pathogenic (Lankester et al. 2007), coinfecting parasite species may have contributed to calf mortality in this study, presumably due to their combined impacts on host immune function and body condition. This is consistent with the expectation that meningeal worm may be one factor important for the southern range contractions of moose (Feldman et al. 2017) and evidence that coinfections exacerbated calf mortality from winter ticks in Vermont (DeBow et al. 2021). By including the number of additional internal parasite species detected, we demonstrated the identity of the parasite species among *P. tenuis* , *N. caninum*, *Nematodirus* spp., and *Moniezia* spp. mattered little; rather, the additional stress of parasite coinfection may have exacerbated death from giant liver fluke regardless of parasite species. Yet, causation and mechanisms behind this association are unclear; we could not assess whether poor body condition predisposed moose to parasite infection or vice versa. Additionally, freezing of fecal samples may have destroyed parasite eggs and reduced our prevalence estimate for the sample (Schurer et al. 2014); thus, our coinfection estimate is a minimum, and the importance of parasites may be even more important than observed. Continued parasite investigations could help determine whether the pattern of increasing mortality risk is consistent with different suites of parasites or varies with parasite intensity (Park and Ezenwa 2020). Additionally, winter ticks were not included as a coinfecting parasite species in our analysis considering their presence on every captured calf moose and comparatively light infestations relative to those that induce epizootics (Grauer 2024; DeBow et al. 2021). However, numbers of ticks, as well as their associated pathogens (Elliott et al. 2021), could be included in investigations of parasite intensity, considering the energetic costs of tick infestation (Musante et al. 2007) and potential for increasing infestations as the severity and length of winters decrease and moose density increases (DeCesare et al. 2024). Investigating the interactions between various disease‐causing agents within moose would improve our understanding of mechanisms that underpin susceptibility to multiple parasite infection and their cumulative negative effects.

Nutritional energy available to moose was included repeatedly in our top models for calf mortality risk but was only marginally significant, with no significance in the univariate nutrition model. Nutritional condition has been correlated with diminished reproduction and survival in adult moose (Oates et al. 2021) and could similarly influence resistance and resilience to parasite infections (Ezenwa 2004). The trend of effect in our multivariate top model was positive, suggesting adequate nutrition could act as a buffer to minimize mortality when moose are faced with fluke and coinfecting parasite infections. Furthermore, inadequate nutrition can be exacerbated by parasite coinfection through resource competition and immune‐mediated interactions (Ezenwa 2021). Forage quality and quantity currently appear to be adequate for the moose population in New York (Peterson et al. 2022) but could become a limiting factor as the population approaches carrying capacity (Kramer et al. 2022). Although the direct impact of white‐tailed deer on moose appears minimal, it is unclear whether deer selection of regenerating forests could contribute to moose nutritional limitation in the future. Therefore, forest management that increases energy available to moose in core areas may be an important management consideration that addresses one of multiple regional stressors to moose.

While we expected thermal stress to influence calf mortality, we found little evidence that ambient temperatures above thermal stress thresholds contributed to mortality risk in this population. Findings from investigations of moose responses to thermal stress have been mixed; for example, ambient temperature was variably important for moose survival in Minnesota depending on the method of analysis (Lenarz et al. 2009; Mech and Fieberg 2014). Studies have repeatedly demonstrated changes in habitat use in response to thermal stress, with moose reducing movement rates and increasing use of thermal refuges including mature conifer and aquatic habitats (Broders et al. 2012; van Beest et al. 2012). Moose have persisted and expanded into areas of high putative thermal stress along their southern range by adapting behaviorally (Wattles et al. 2018), and the biological relevance of published critical thresholds has been questioned (Montgomery et al. 2019). Even though temperature alone may have variable effects on moose survival, heat stress could indirectly, via moose habitat selection, increase the risk of acquiring giant liver fluke or other parasite infections. Shifting habitat use has also been implicated in moose seasonal mass change, reflecting trade‐offs of selecting habitat for forage quality versus thermoregulation (van Beest and Milner 2013). These synergies may have cascading impacts on body condition, parasite infection, and ultimately, survival and population growth.

While survival of moose in New York is primarily limited by giant liver fluke, the potential population‐level impacts of parasite‐induced disease are unknown. If disease incidence increases or disease‐induced mortality is additive to other sources of mortality, population growth rates may decline in the future. Rates of giant liver fluke infection in moose may increase as changing climate promotes overlap with white‐tailed deer (Weiskopf et al. 2019) and enhances parasite transmission from gastropod intermediate hosts (Marcogliese 2008). In addition to impacting survival, the negative impacts of parasites and disease on body condition can translate to declines in fecundity (Albon et al. 2002), delaying age of first reproduction, reducing twining rates, and reducing calf body size (Tiilikainen et al. 2012). Successful reproduction is consistently correlated with biomass in cervids, where body condition and pregnancy rates decline with increasing parasite burden (Hughes et al. 2009). For example, nematode infections in Dall's sheep ( *Ovis dalli* ) decreased adult female body condition and altered sex ratios of fetuses (Aleuy et al. 2020). Parasites and their subsequent diseases impact the energy budgets of hosts through a variety of pathways, with ranging effects on individual fitness. This work highlights the importance of cumulative stressors on susceptible juveniles and necessitates further investigation of the population‐level effects of coinfection.

Considering the important impact of parasites on moose survival, management strategies that reduce parasite infection in moose could minimize mortality risk in this population. Specifically, as both *F. magna* and *P. tenuis* presence in the region requires white‐tailed deer primary hosts, reducing deer overlap with moose may be necessary to lower moose infection risk and subsequent mortality. Additional research could help elucidate coinfection dynamics within moose hosts and determine whether specific coinfecting parasite species are particularly destructive, and whether or to what extent adequate resources buffer against the deleterious effects of parasites. Continued monitoring of moose survival could also allow assessment of how these dynamics interact with changing climatic conditions and impact future moose population persistence.

## Author Contributions

**Jennifer A. Grauer:** conceptualization (equal), formal analysis (lead), visualization (lead), writing – original draft (lead). **Jacqueline L. Frair:** conceptualization (equal), funding acquisition (equal), project administration (equal), resources (equal), writing – review and editing (equal). **Krysten L. Schuler:** conceptualization (equal), resources (equal), writing – review and editing (equal). **Manigandan Lejeune:** formal analysis (supporting), writing – review and editing (equal). **David W. Kramer:** resources (supporting), writing – review and editing (equal). **Angela K. Fuller:** conceptualization (equal), funding acquisition (lead), project administration (lead), resources (equal), writing – review and editing (equal).

## Funding

This study was supported by the U.S. Fish and Wildlife Service, Wildlife Restoration Grant W‐178‐R.

## Conflicts of Interest

The authors declare no conflicts of interest.

## Supporting information


Appendix S1

## Data Availability

The data that support the findings of this study are openly available at https://doi.org/10.5066/P13MZ4WZ (Grauer and Fuller 2025).
