# Supplementary material for: Moose Survival and Habitat‐Associated Risk of Endoparasites
Source: Ecol Evol. 2025 Dec 29;15(12):e72721. doi: 10.1002/ece3.72721 (PMC12746053; doi:10.1002/ece3.72721)
Supplement: Supplementary file 1 — Appendix S1 [file ECE3-15-e72721-s001.docx]

Supporting Information for Moose survival and habitat associated risk of endoparasites

Jennifer A. Grauer^1^, Jacqueline L. Frair^2^, Krysten L. Schuler^3^, Manigandan Lejeune^4^, David W. Kramer^5^, Angela K. Fuller^6^

^1^ New York Cooperative Fish and Wildlife Research Unit, Department of Natural Resources and the Environment, Cornell University, 226 Mann Drive, Ithaca, NY 14853, USA

^2^ Department of Environmental Biology, SUNY College of Environmental Science and Forestry, 1 Forestry Drive, Syracuse, NY 13210, United States

^3^ Wildlife Health Lab, Public and Ecosystem Health Department, College of Veterinary Medicine, Cornell University, 240 Farrier Road, Ithaca, NY 14853, USA

^4^Department of Population Medicine and Diagnostic Sciences, Cornell University College of Veterinary Medicine, Ithaca, New York, USA, [ml872@cornell.edu](mailto:ml872@cornell.edu)

^5^ Roosevelt Wild Life Station, SUNY College of Environmental Science and Forestry, Syracuse, NY 13210, USA, David.Kramer@dec.ny.gov

^6^ U.S. Geological Survey, New York Cooperative Fish and Wildlife Research Unit, Department of Natural Resources and the Environment, Cornell University, 226 Mann Drive, Ithaca, NY 14853, USA

*Any use of trade, firm, or product names is for descriptive purposes only and does not imply endorsement by the U.S. Government.*

Appendix S1. Competing Model Set with Cumulative Nutrition

In order to explore the covariate of cumulative nutrition available to moose (*Alces alces*) within our Cox proportional hazards models for calf survival, we created a second model set that included cumulative nutrition, monthly giant liver fluke (*Fascioloides magna*) infection risk, temperature, and number of coinfecting parasites. Because the cumulative structure of our fluke risk and nutrition covariates led to increasing values for an individual moose over the time it was included in the study these covariates were collinear and could not be included in the same model set.

Here we provide details on the model set following the same structure as included models, where cumulative fluke risk is replaced by monthly fluke risk and monthly nutrition is replaced by cumulative nutrition. This allowed us to explore whether compounding nutritional availability for a given moose affected moose mortality when compared to our original model set.

All included covariates had a correlation of |r| < 0.56, with monthly fluke risk and cumulative nutrition having the highest correlation. The global model met assumptions for independence of the model and individual covariates (global Χ^2^= 5.20, p= 0.64). Ten models had ΔAIC_c_<2 (Table S2), and the top-ranked model included the additive effects of monthly fluke risk and temperature (Table S3). Both covariates were significant at an alpha of 0.1 but not 0.05 (p=0.08), with fluke risk positively affecting mortality, and temperatures closer to critical stress thresholds associated with higher mortality. Additional competing models included the null model, univariate models for the effects of coinfecting parasites, temperature, and fluke risk, 3-way additive models, and the interactive effects of temperature and parasites with fluke risk. The global model was ranked lowest and included no statistically significant covariates for monthly survival.

Table S1. Energy value per km^2^ used for habitat cover types in the Adirondack Park (Ferree & Anderson 2013; Kramer et al. 2022) across seasons, estimated as animal use days (AUD) by Peterson et al. (2022). AUD estimates accounted for the energetic requirements of adult female moose (*Alces alces*) in different seasons and the digestible energy and crude protein available in vegetation. Values were multiplied by monthly mean proportions of each cover type within 115 m circular buffers around moose relocations to characterize nutritional energy available to each moose.

| **Cover Type** | **Summer Energy**  **(May-October)** | **Winter Energy**  **(November-April)** |
| --- | --- | --- |
| Conifer forest | 0.0048 | 0.1351 |
| Upland deciduous/mixed | 0.0070 | 0.0316 |
| Lowland deciduous/mixed | 0.0071 | 0.1584 |
| Wooded wetland | 0.0591 | 0.1593 |
| Open wetland | 0.0057 | 0.2654 |
| Regenerating forest | 0.0561 | 0.0023 |

Table S2. Model selection of Cox proportional hazards for monthly survival of calf moose (*Alces alces*) in the Adirondack Park, New York, USA. This alternative model set includes monthly giant liver fluke (*Fascioloides magna*) infection risk, cumulative nutrition available, number of coinfecting parasites, and temperature (temp). Included are the additive and interactive models tested with their number of parameters (k), Akaike’s information criterion corrected for small sample size (AIC_c_), and difference in AIC_c_ (ΔAIC_c_).

| **Model** | **k** | **AIC_c_** | **ΔAIC_c_** |
| --- | --- | --- | --- |
| fluke + temp | 3 | 66.048 | 0 |
| parasite | 2 | 66.280 | 0.232 |
| fluke * temp | 4 | 66.501 | 0.453 |
| temp | 2 | 66.673 | 0.625 |
| fluke + parasite + temp | 4 | 66.695 | 0.647 |
| null | 1 | 66.839 | 0.791 |
| fluke + parasite | 3 | 66.843 | 0.795 |
| fluke | 2 | 67.070 | 1.022 |
| fluke * parasite | 4 | 67.571 | 1.523 |
| fluke + temp + nutrition | 4 | 68.047 | 1.999 |
| nutrition | 2 | 68.311 | 2.263 |
| fluke + parasite + nutrition + temp | 5 | 68.612 | 2.564 |
| fluke + parasite + nutrition | 4 | 68.850 | 2.802 |
| fluke * nutrition | 4 | 68.867 | 2.819 |
| fluke + nutrition | 3 | 69.025 | 2.977 |
| full | 8 | 71.819 | 5.771 |

Table S3. Parameter values for top alternate Cox proportional hazards model set of calf moose (*Alces alces*) mortality in New York, USA from 2022-2024 that included monthly giant liver fluke (*Fascioloides magna*) infection risk and difference between ambient and critical stress temperature thresholds. Included are the coefficient (β) values, hazard ratio (Exp(β)), 95% confidence intervals (CI), and statistical significance (P) for each included covariate.

| **Covariate** | **β** | **Exp(β)** | **95% CI** | **P** |
| --- | --- | --- | --- | --- |
| Fluke risk | 0.49 | 1.63 | 0.93-2.85 | 0.08 |
| Temperature | -6.23 | 0.002 | <0.01-2.13 | 0.08 |

Literature Cited

Ferree, C., & Anderson, M. G. (2013). A map of terrestrial habitats of the northeastern United States: methods and approach. The Nature Conservancy, Eastern Conservation Science, Eastern Regional Office. Boston, MA.

Kramer, D. W., Prebyl, T. J., Nibbelink, N. P., Miller, K. V., Royo, A. A., & Frair, J. L. (2022). Managing moose from home: determining landscape carrying capacity for Alces alces using remote sensing. Forests, 13(2), 150.

Peterson, S., Kramer, D., Hurst, J., Spalinger, D., & Frair, J. (2022). Forage and habitat limitations for moose in the Adirondack Park, New York. Alces, 58, 1-30.
